# Editorial: Epigenetic regulation of autophagy in inflammatory diseases

**DOI:** 10.3389/fimmu.2024.1387459

**Published:** 2024-03-05

**Authors:** Kai Wang, Chao Yang, Bailong Tao, Shicheng Guo, Haiyong Wang

**Affiliations:** ^1^ Key Laboratory of Epigenetics and Oncology, The Research Center for Preclinical Medicine, Southwest Medical University, Luzhou, China; ^2^ National Engineering Research Center for Marine Aquaculture, Institute of Innovation & Application, Zhejiang Ocean University, Zhoushan, China; ^3^ Laboratory Research Center, The First Affiliated Hospital of Chongqing Medical University, Chongqing, China; ^4^ University of Wisconsin-Madison, Madison, WI, United States; ^5^ Department of Internal Medicine Oncology, Shandong Cancer Hospital and Institute, Shandong First Medical University and Shandong Academy of Medical Sciences, Jinan, China

**Keywords:** epigenetic regulation, autophagy, inflammatory diseases, SIRTUIN, M^6^A modification

Autophagy is a conserved stress response mechanism that occurs in eukaryotes. It involves the degradation of various cargoes (e.g., damaged organelles, misfolded proteins, etc.) within autolysosomes and is regulated by autophagy-related genes (Atgs). Autophagy helps maintain homeostatic balance and regulates physiological functions. Many human diseases are accompanied by inflammation. Autophagy clears pathogens, inhibits the production of inflammatory factors, and regulates the function of immune cells, reducing inflammatory response (1). Dysregulation of autophagy can contribute to the development and progression of various diseases, including inflammatory diseases (ID) (2).

Epigenetic regulation or the regulation of gene expression through chromatin structure alteration and DNA methylation impacts cellular function and physiological states (3). Increasing evidence has revealed the interaction between autophagy, inflammation, and epigenetic regulation (4).

Herein, we aimed to shed light on the role of autophagy and its epigenetic modifications in ID, highlighting the clinical implications of targeting epigenetics and/or autophagy for the prevention and treatment of these pathologies. To this end, this Research Topic includes 11 research articles and reviews.

Using gene enrichment analysis, Cao et al. screened differentially expressed genes (DEGs) from a public RNA-seq dataset and identified four immune- and metabolism-related hub genes (CD40LG, MAPK14, CD28, S100A12), providing new insights into postoperative systemic inflammatory dysregulation. In the past decade, Lin et al. systematically analyzed the regulatory role of autophagy in lung diseases, deepening our understanding of their pathogenesis. Na^+^/K^+^-ATPase, a major driver of Na^+^ transport in alveolar type II epithelial cells, was shown to promote alveolar fluid clearance (5). Na^+^/K^+^-ATPase is degraded during autophagy (Wen et al.). Furthermore, using a lipopolysaccharide-induced inflammation mouse model, Wen et al. revealed that insulin upregulated Na^+^/K^+^-ATPase expression by inhibiting autophagy, leading to a reduction in the inflammatory response. An original study reported by Wang et al. revealed that autophagy-mediated endocytosis of natural killer (NK) group 2D receptors on the surface of NK cells and their lysosomal degradation exacerbates radiation-induced pneumonia.

Autophagy and inflammation studies have identified potential targets for anti-osteoarthritis (OA) therapy. Using relevant datasets derived from Gene Expression Omnibus and autophagy databases, Qin et al. identified four Atgs (MAP1LC3B, CDKN1A, MYC, DDIT3) associated with inflammation/immunity. In addition, Lu et al. reported a heritability study linking OA and spondylitis.

Inflammation and epigenetic regulation mediated through specific factors impact autophagy, affecting the development of inflammation. Yang et al. revealed that the m^6^A demethylase, fat mass and obesity-associated protein, regulated autophagy and renal fibrosis by impairing the stability of p62 mRNA. Zhang et al. described how sirtuins, histidine deacetylases, and related co-substrates regulate autophagy and macrophage polarization, impacting glucose metabolism. They also reported that sirtuins regulate autophagy by deacetylating autophagy-related proteins, suggesting the potential use of sirtuin modulators in tuberculosis therapy (Zhang et al.). A review article by Mao et al. described the epigenetic regulation of pulmonary hypertension during autophagy, including acetylation signaling of autophagy, methylation of histones and DNA, and RNA alternative splicing.

These data on targeting autophagy and epigenetic modifications are crucial for understanding how epigenetic modifications influence autophagy mechanisms. They could potentially offer personalized treatment strategies for patients with various diseases, including pulmonary hypertension. Additionally, Jiang et al. discuss how programmed cell death, including autophagy, affected epigenetic regulation in hypoxic-mediated pulmonary hypertension.

Studies on drug regulation of autophagy and epigenetics offer new treatment targets for ID. Canagliflozin, a sodium-glucose cotransporter-2 inhibitor approved by the Food and Drug Administration for the treatment of diabetes, targeted the epigenetic modifiers histone deacetylases 6 and 2, inhibiting the progression of tumors (6, 7) and activated autophagy yielding anti-inflammatory effects (8). Canagliflozin attenuated renal fibrosis *in vitro* and *in vivo* through an autophagy-mediated m^6^A modification (Yang et al.).

Obeticholic acid is a specific ligand for farnesoid X receptor (FXR), which is regulated by various epigenetic modifications such as methylation and acetylation (9). It targets the Toll-like receptor 4/transforming growth factor-beta 1/autophagy pathway mitigating non-alcoholic fatty liver disease (10). Using single-cell RNA-seq data, Gou et al. identified CXCL16 as a DEG in NKT cells, exploring the potential of targeting the chemokine (C-X-C motif) ligand (Cxcl)16/CXCR6 pathway. Furthermore, co-treatment with obeticholic acid and 5β-cholanic acid 3 inhibited the malignant progression of hepatocellular carcinoma in an *in situ* carcinoma mouse model (Gou et al.). However, how obeticholic acid-regulated autophagy affects tumor epigenetic modifications still needs to be explored in depth.

This work covers just a fraction of the evolving field of epigenetic regulation of autophagy, which is rapidly advancing. In summary, understanding the interplay between epigenetic regulation and autophagy is crucial for deciphering the pathogenesis of ID and identifying novel treatment targets and strategies.

## Author contributions

KW: Funding acquisition, Supervision, Validation, Writing – original draft, Writing – review & editing. CY: Writing – review & editing. BT: Writing – review & editing. SG: Writing – review & editing. HW: Writing – review & editing.
